# Analysis of the risk factors and clinical features of Mycoplasma pneumoniae pneumonia with embolism in children: a retrospective study

**DOI:** 10.1186/s13052-022-01344-0

**Published:** 2022-08-20

**Authors:** Chunjiao Han, Tongqiang Zhang, Jiafeng Zheng, Peng Jin, Qi Zhang, Wei Guo, Yongsheng Xu

**Affiliations:** 1 Clinical School of Paediatrics, Tianjin Medical University, Tianjin, China; 2 Department of Pulmonology, Tianjin Children’s Hospital (Children’s Hospital of Tianjin University), Tianjin, China

**Keywords:** *Mycoplasmapneumoniae* pneumonia, Embolism, Children, Risk factors, Clinical characteristics

## Abstract

**Background:**

*Mycoplasma* *pneumoniae* pneumonia (MPP) is a prevalent disease in community-acquired pneumonia among children. However, in addition to respiratory manifestations, it may also develop extra-pulmonary complications. Embolism is one of the uncommon extra-respiratory manifestations prone to severe sequelae and even death. This study aims to analyze the clinical features of MPP with embolism in children, and explore the associated risk factors of embolism in MPP patients.

**Methods:**

A retrospective case–control analysis was performed on 48 children with MPP admitted to our hospital wards between January 2010 and December 2021. Embolism group comprised children with embolism by CTA or MRA results, whereas the non-embolism group comprised children with clinical suspicion of embolism but negative diagnostic imaging support. The clinical features, laboratory findings and imaging were analyzed to explore the risk factors for embolism in children with MPP.

**Results:**

A total of 48 children with MPP were enrolled in the study (16 cases and 32 controls). In the embolism group, 10 patients (62.5%) had pulmonary embolism, 3 patients (18.75%) presented ventricle embolism, 2 patients (12.5%) presented cerebral and carotid artery embolism, one patient (6.25%) had a cerebral embolism, limb, and spleen, respectively. The univariate analysis revealed the maximum body temperature (Tmax), CRP, D-dimer (closest to CTA/MRA), the percentage of neutrophils (N%), pulmonary consolidation (⩾ 2/3 lobe), pleural effusion and atelectasis have significant differences between the embolism group and non-embolism group (*P* < 0.05). Multivariate logistic regression analysis showed that D-dimer (closest to CTA/MRA) > 3.55 mg/L [OR = 1.255 (95% CI: 1.025—1.537), *P* < 0.05], pulmonary consolidation (⩾ 2/3 lobe) [OR = 8.050 (95% CI: 1.341—48.327), *P* < 0.05], and pleural effusion [OR = 25.321 (95% CI: 2.738—234.205), *P* < 0.01] were independent risk factors for embolism in children with MPP.

**Conclusion:**

In conclusion, MPP with embolism patients have more D-dimer values and severe radiologic manifestations.

## Background

*Mycoplasma pneumoniae* pneumonia (MPP) is a prevalent and self-limiting disease in the community-acquired pneumonia in children [1]. However, in addition to respiratory manifestations, it may also develop extra-pulmonary complications, including the cardiovascular system, digestive organ, neurological, dermatological manifestations, etc [2]. Embolism is one of the uncommon extra-respiratory manifestations that has been reported in previous studies [3, 4]. It can present in nearly any part of the body, specifically in the lungs, then in the limbs [3]. Embolism is prone to severe sequelae and even life-threatening if not timely diagnosed and treated. In this study, we retrospectively analysed 16 children in MPP with embolism received in our hospital between January 2010 and December 2021. Additionally, we compared basic information, clinical manifestations and imaging results between 16 children in MPP with embolism as well as 32 children with MPP who had a clinical suspicion of embolism but negative radiological results. We aimed to explore the related factors predicting MPP with embolism that help clinicians in early detection and treatment.

## Methods

### Patients and definitions

A total of 16 children in MPP with embolism were admitted to the Respiratory Department of Tianjin Children’s Hospital between January 2010 and December 2021. Further, 32 children in MPP with clinical suspicion of embolism were randomly selected within the same period. The embolism and non-embolism groups were divided based on whether there was embolism underwent CTA/MRA. The inclusion criteria for the embolism group were as follows: ① The condition met the diagnostic criteria for Mycoplasma pneumoniae pneumonia: (i) positive results for the serological test (MP-IgM positive and antibody titer ≥ 1:160); (ii) the positive results for MP polymerase chain reaction (PCR) tests of nasopharyngeal secretions. ② The condition met the diagnostic criteria for embolism: CTA/MRA indicates embolism in the blood vessel or heart. ③ Informed consent was signed for CTA/MRA examination. The inclusion criteria for the non-embolism group were as follows: ① Symptoms including shortness of breath, dyspnea, pain or weakness in any part of the body. ②Severe laboratory indicators such as inflammatory markers and coagulation function. ③ Severe imaging findings such as massive pneumonia consolidation, pleural effusion and necrotizing pneumonia. The exclusion criteria were as follows: ① Combined with other pathogenic infections. ② Basic diseases, including chronic cardiopulmonary disease, rheumatism, immune deficiency disease, or severe blood system disease. ③ The age was more than 15 years old. All procedures performed in studies were following the Ethics Committee of Tianjin Children’s Hospital. All patients’ data were analyzed anonymously.

### Etiological detection

All the children completed the following detection within 24 h of admission: MP-DNA detected by MP polymerase chain reaction (PCR) tests by nasopharyngeal swab, phlegm respiratory pathogen eight (influenza A and influenza B, respiratory syncytial virus, adenovirus, metapneumovirus, parainfluenza virus type 1, 2, 3), blood respiratory pathogen IgM antibody nine (eosinophilic lung Legionella bacteria, *Mycoplasma* pneumoniae, Chlamydia, Rickettsia, adenovirus pneumonia, syncytial virus, influenza A virus, influenza B virus, and parainfluenza), tuberculosis and bacterial culture.

### Data collection

The clinical data of all children were collected as following: (1) basic information: name, gender, age, BMI, time to CTA/MRA (days), previous venous thrombo embolism (VTE), cardiovascular diseases (CVDs) and ICU admission; ⑵ clinical manifestations: Tmax, heart rate, main symptoms, distribution of embolism and outcome. ⑶ laboratory tests: routine blood tests, inflammatory markers, blood biochemistry, autoantibody, coagulation function and humoral immunity. ⑷ imaging examination results.

### Modified PIRO severity score

The modified PIRO severity score [5] comprised predisposition (malnutrition), insult (complicated chest radiograph), response (hypoxemia, hypotension, CRP > 0.5 mg/dL, PCT > 0.5 ng/dL), and organ dysfunction. These significant variables were given a value of 0 (for those who were not at risk) and 1 (for those at risk). A scale of 0–7 modified PIRO severity score was stratified into three levels of risk: low (≤ 2), moderate (3-4), and high (5-7).

### Statistical analysis

Statistical analyses were conducted in SPSS 24.0. The normal distribution data was represented by mean ± standard deviation (x ± s). The independent sample t test was used for comparison. The non-normal distribution was described as median (P25, P75), which comparisons were made by the Mann–Whitney U-test. And the chi-square test was used for categorical data. Logistic regression analysis was performed to examine significant risk factors in the univariate analysis. Receiver operating characteristic (ROC) curves were used to identify potential markers of children in MPP with acute embolism. *P* < 0.05 was considered statistically significant.

## Result

### Basic information

A total of 48 MPP children were included in this study. There were 16 (33.3%) cases in the embolism group (6 male; 10 female) and 32 cases (66.7%) in the non- embolism group (21 male; 11 female). The mean age of embolism and non- embolism patients was 7.13 ± 2.94 years and 6.08 ± 3.11 years, respectively. One child had obesity. There was no significant difference in age, BMI and gender between the two groups (*P* > 0.05).

### Modified PIRO severity score

Based on the modified PIRO severity score, half of the patients (2 patients with 4 points and 6 patients with 3 points) were at medium risk, whereas the other half (8 patients with 2 points) were at low risk in the embolism group. In the non- embolism group, 13 patients (3 patients with 4 points and 10 patients with 3 points) were at medium risk, whereas 19 (15 patients with 2 points, 4 patients with 1 points) were at low risk in the non-embolism group. There was no significant difference in the severity score between the two groups (*P* > 0.05).

### Clinical manifestations

All patients had a cough. All patients with embolism and 31 (97%) patients without embolism had a fever. And the MPP with embolism group had a higher fever than that of the non- embolism group (*P* < 0.05). All patients had no history of recent surgery, previous VTE and CVDs. One patient had been to ICU in embolism group and non- embolism group respectively. Six patients (37.5%) had more than one distribution of embolism (Table 1). In embolism group, 10 patients (62.5%) had pulmonary embolism, 3 patients (18.75%) had ventricle embolism, 2 patients (12.5%) presented cerebral and carotid artery embolism, one patient (6.25%) presented cerebral embolism, limb and spleen respectively. There was no significant difference in age, gender, BMI and time to CTA between the two groups (Table 2).

**Table 1:** The clinical symptoms and relevant involved vessels of pediatric MPP-associated embolism

Case	Age	Main symptoms	Lung consolidation site	Embolic site	Antibody
1	2	Fever, Cough	Lower lobe of left lung	Left lower pulmonary artery	ACA(-); ANA(-)
2	5	Fever, Cough, Shortness of breath	Both lung	Bilateral inferior pulmonary arteries	*NA*
3	7	Fever, Cough	Upper lobe of right lung	Right lower pulmonary artery	*NA*
4	5	Fever, Cough, Hypoxemia	Both lung	Right lower pulmonary artery, Right ventricle	*NA*
5	3	Fever, Cough, Weakness of both lower limbs and Unequal height of shoulders	Both lung	Left pulmonary artery trunk, Left superior and inferior pulmonary artery, Internal jugular vein	ANA(-)
6	8	Fever, Cough	Lower lobe of left lung	Bilateral inferior pulmonary arteries	ANA(-)
7	8	Fever, Cough, Neck pain, Shortness of breath, Hypoxemia	Lower lobe of right lung	Left lower pulmonary artery, Right ventricle	*NA*
8	8	Fever, Cough, Intermittent sword process pain	Upper lobe of both lung, Lower lobe of left lung	Right lower pulmonary artery	*NA*
9	9	Fever, Cough, Hypoxemia, Dyspnea	Lower lobe of both lung	Right inferior pulmonary artery	ANA( +), titer 1:100; cANCA(-); Protein C activity 179.2%(70–140); Protein S activity 59.5%(70–123)
10	7	Fever, Cough, Occasional left chest pain	Both lung, Lower lobe of right lung, Left lung	Left upper pulmonary artery, Right inferior pulmonary artery	*NA*
11	7	Fever, Cough, The muscle tension of the right limb decreased, and the muscle strength was grade 4	Upper lobe of right lung, Left lung	Left internal carotid artery, Middle cerebral artery	ANA(-)
12	9	Fever, Cough, Right limb dyskinesia, positive right Babinski sign, positive double heel knee reflex	Left lung, Lower lobe of right lung	Left anterior cerebral artery, Middle cerebral artery deep perforator infarction	*NA*
13	12	Fever, Cough, Right lower limb pain, The skin of the right foot bloom Reduced skin temperature, and weakened pulse of the right dorsal foot artery	Both lung	Right lower limb artery embolization (right external iliac artery, right deep femoral artery, right posterior tibial artery), Right ventricle	ACA-Ab IgG27.4CU/ml, ACA-Ab IgM2.7CU/ml; ACA-Ab IgA5.1CU/ml; Protein C activity 110.5%(70–140); Protein S activity 49.5%(70–123)
14	3	Fever, Cough, Left limb weakness, Dyspnea	Left lung	Brain (right frontotemporal parietal and right basal ganglia), right internal carotid artery, right middle cerebral artery, right anterior cerebral artery	*NA*
15	9	Fever, Cough, Left axillary rib pain, shortness of breath, Blood in sputum	Right lung	Right ventricle	ANA( +), titer 1:100
16	12	Fever, Cough, Intermittent abdominal pain and vomiting	Left lung	Splenic artery	*NA*

*ACA* Anti-cardiolipin antibody, *ANA* Anti-nuclear antibody

**Table 2:** Clinical characteristics and laboratory values of embolism in children with MPP

Characteristics	Total (*n* = 48)	Embolism (*n* = 16)	Non-Eembolism (*n* = 32)	*P* value
Age (year)	6.43 ± 3.06	7.13 ± 2.94	6.08 ± 3.11	0.270
Proportion of male	21/48	10/16	11/32	0.064
BMI	16.24 (14.79, 18.90)	17.20 (15.14, 20.22)	15.64 (14.63, 18.20)	0.182
Tmax (℃)	40.00 (39.30, 40.00)	40.00 (39.78, 40.38)	39.80 (39.00, 40.00)	0.064
Heart rate	107.19 ± 11.85	108.5 ± 7.95	106.53 ± 13.46	0.593
Time from onset to admission (day)	10.60 ± 3.75	10.38 ± 3.72	10.72 ± 3.82	0.768
Time from onset to CTA/MRA (day)	13.77 ± 6.37	13.69 ± 4.81	13.81 ± 7.09	0.943
Recent surgery	0	0	0	
Previous VTE	0	0	0	
CVDs	0	0	0	
ICU admission	2 (0.04%)	1 (0.06%)	1 (0.03%)	
Recurrence	0	0	0	
Death	0	0	0	
D-dimer (closest to CTA/MRA)	2.35 (0.43, 6.86)	6.25 (3.98, 9.30)	1.05 (0.13, 3.30)	0.002
D-dimer (hospital admission)	1.40 (0.29, 3.88)	2.20 (0.63, 4.52)	1.15 (0.10, 1.95)	0.039
APTT	30.31 ± 6.00	29.97 ± 5.25	30.48 ± 6.42	0.786
PT	11.85 (10.93, 12.68)	11.85 (10.95, 12.48)	11.85 (10.93, 12.88)	0.686
WBC (closest to CTA/MRA)	10.37 (7.26, 13.62)	10.91 (8.62, 13.57)	9.83 (6.99, 14.04)	0.320
WBC (hospital admission)	9.04 (7.10, 13.98)	13.58 (8.17, 15.80)	8.40 (6.63, 12.64)	0.029
Fg (closest to CTA/MRA)	3.47 ± 1.00	4.09 ± 1.15	3.16 ± 0.75	0.001
Fg (hospital admission)	3.83 (3.11, 4.69)	4.42 ± 1.54	3.71 ± 1.06	0.067
PLT	328.44 ± 128.46	347.06 ± 80.75	319.13 ± 146.96	0.400
CRP	47.05 (8.78, 89.28)	87.00 (48.20, 139.75)	16.30 (7.45, 62.75)	0.001
N%	76.27 (64.39, 81.78)	79.30 (73.75, 88.45)	72.60 (58.55, 79.08)	0.025
IL-6	34.37 (7.94, 136.00)	28.20 (14.72, 64.79)	46.68 (7.72, 157.55)	0.630
PCT	0.15 (0.07, 0.29)	0.17 (0.05, 0.29)	0.15 (0.07, 0.27)	0.094
FER	227.05 (121.68, 539.43)	323.45 (186.75, 582.60)	172.70 (90.87, 436.28)	0.120
CK	90.00 (46.25, 122.75)	58.00 (33.50, 164.50)	98.00 (70.25, 122.75)	0.571
CKMB	2.00 (2.00, 5.00)	2.00 (2.00, 4.75)	2.00 (2.00, 5.00)	0.974
LDH	518.50 (373.75, 717.75)	668.00 (528.50, 869.75)	435 (342, 674.25)	0.020
ALT	18.00 (12.00, 35.75)	18.00 (15.00, 49.50)	17.50 (11.00, 35.75)	0.358
IgM	1.46 (0.93, 2.14)	2.09 (1.40, 3.08)	1.16 (0.86, 1.96)	0.024

*BMI* Body mass index, *WBC* White blood cell, *N* Neutrophils, *L* Lymphocyte, *CRP* C-reactive protein, *IL-6* Interleukin-6, *FER* Ferritin, *PCT* Procalcitonin, *PT* Prothrombin time, *APTT* Activated partial thromboplastin time, *Fg* Fibrinogen, *ALT* Alanine transaminase, *LDH* Lactate dehydrogenase, *CK* Creatine kinase, *CKMB* creatine kinase-MB, *IgM* Immunoglobulin M

### Laboratory tests

**Fig. 1:**
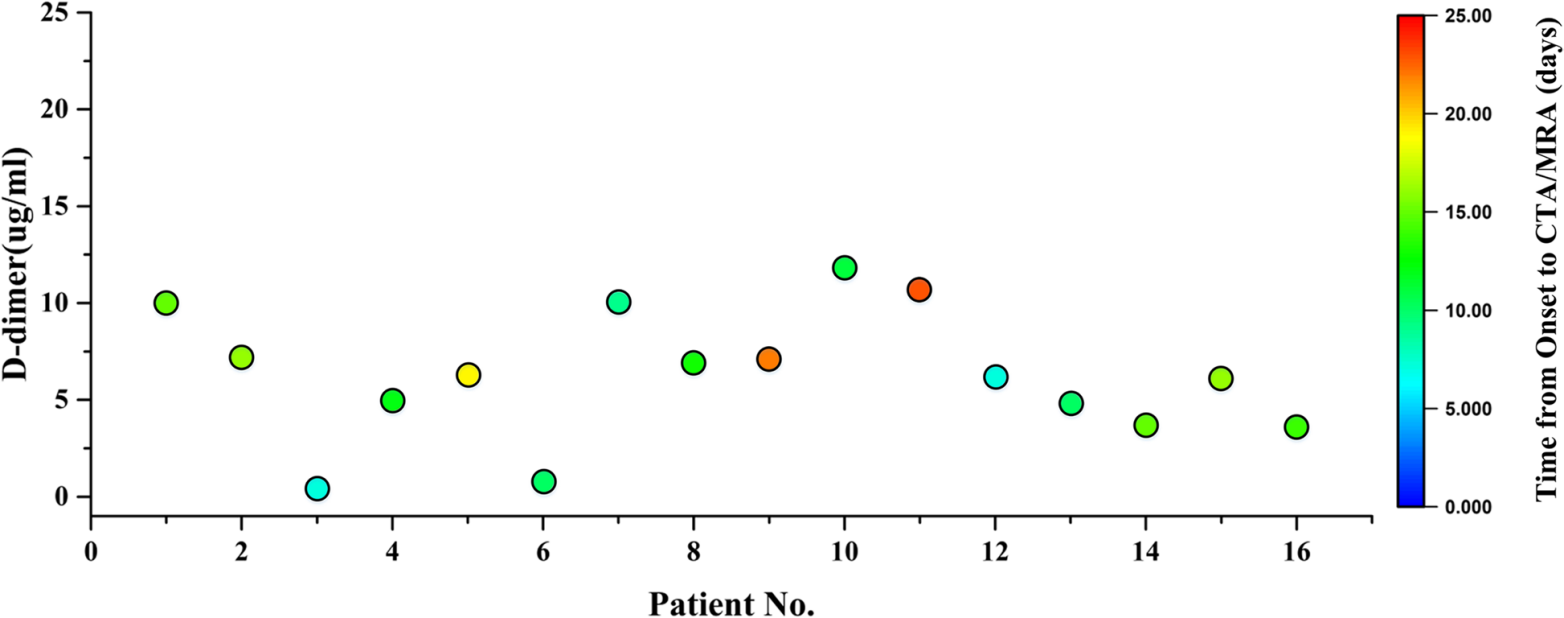
D-dimer values of patients (*n* = 16) tested over multiple days. Color-coded circles correspond to D-dimer values on different dates

In the embolism group, the values of D-dimer (closest to CTA/MRA), D-dimer (hospital admission), WBC (hospital admission), Fg (closest to CTA/MRA), C-reactive protein (CRP), neutrophil percent (N%), lactate dehydrogenase (LDH) and IgM (Immunoglobulin M) were significantly higher than that in the non-embolism group (*P* all < 0.05). The value of APTT in the embolism group was lower than that in the non- embolism group (*P* < 0.05). Nevertheless, PT, WBC (closest to CTA/MRA), Fg (hospital admission), PLT, IL-6, PCT, FER, CK, CKMB and ALT showed no significant difference between the two groups (*P* > 0.05) (Table 2). In the embolism group, two patients were tested for ACA, consequently, one patient was positive, whereas the other was negative. In addition, six patients were tested for ANA, and two patients (33.3%) showed positive (Table 1). In non-embolism group, there were six patients tested for ANA and fourteen patients tested for ACA. All the results were negative. Figure 1 shows the D-dimer values of patients (*n* = 16) tested over multiple days. Color-coded circles correspond to D-dimer values on different dates.

### Imaging examination

**Fig. 2:**
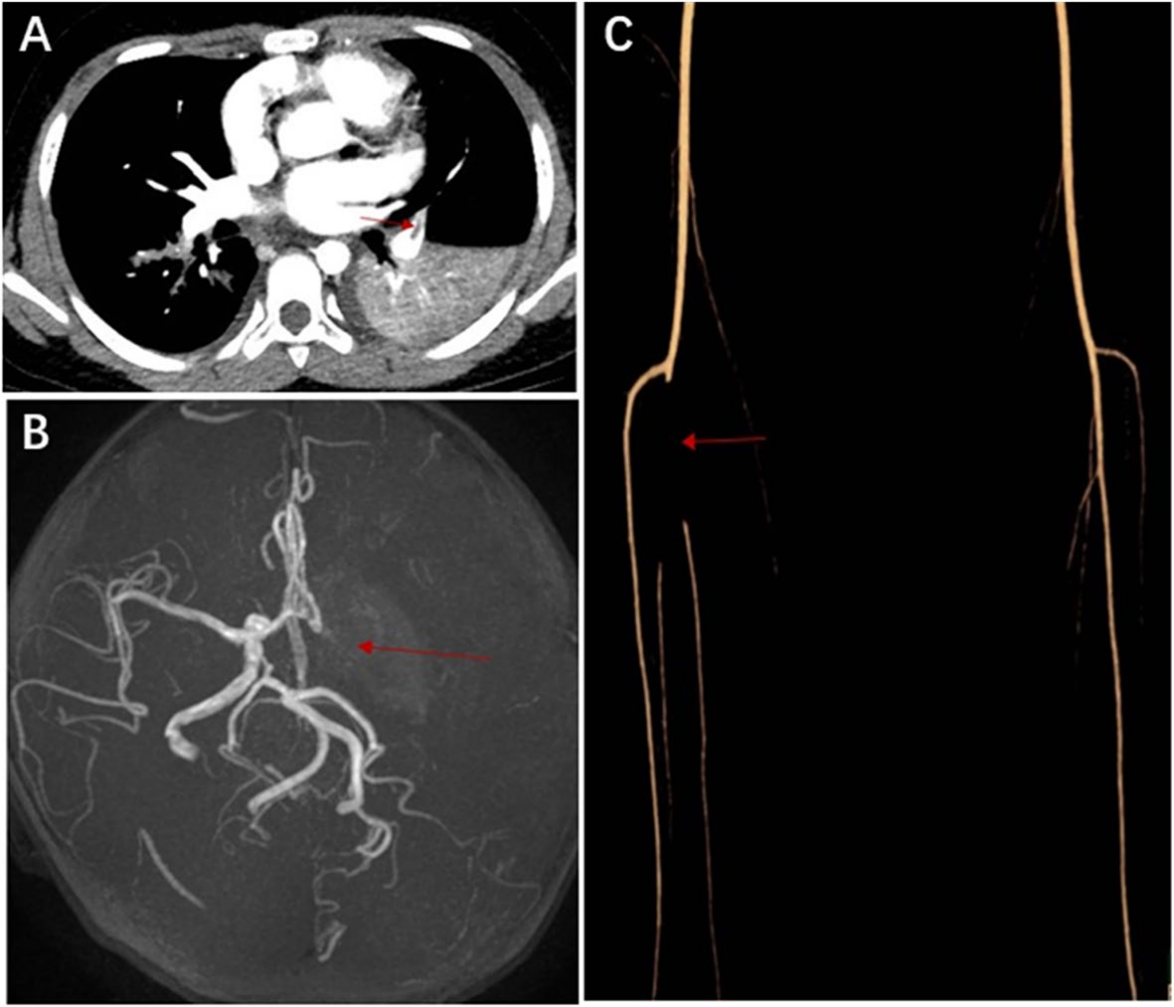
**A** Chest CTA showed a filling defect in the left upper pulmonary artery. **B** MRA examination of the head showed that the cavernous sinus segment of the left internal carotid artery, the middle cerebral artery and its branches were not developed. **C** CTA of lower limbs showed that the right posterior tibial artery was not developed locally

Pulmonary inflammatory consolidation was found in all the patients. Pulmonary embolism occurred on the same side as the pulmonary consolidation in 5 patients. The radiological abnormalities in the embolism-group were more severe than that in non- embolism group (Table 3). There were significant differences in pulmonary consolidation (⩾ 2/3 lobe), pleural effusion and atelectasis between two groups (*P* < 0.01). Nonetheless, the incidence of pleural thickening and necrotizing pneumonia showed no statistical difference between two groups (*P* > 0.05). Figure 2 shows the CTA images of pulmonary embolism, cerebral embolism and lower extremity embolism (Fig. 2).

**Table 3:** Imaging of Embolism and non-Embolism children with MPP

**Radiological features **	**Total (*n*=48) **	**Embolism (*n*=16)**	**Non-Embolism (*n*=32)**	***P* value**
Pulmonary consolidation (⩾ 2/3 lobe)	17 (35.42%)	11 (68.75%)	6 (18.75%)	0.001
Pleural effusion	23 (47.92%)	14 (87.5%)	9 (28.13%)	0.000
Atelectasis	19 (39.58%)	11 (68.75%)	8 (25%)	0.005
Pleural thickening	26 (54.17%)	8 (50%)	18 (56%)	0.682
Necrotizing pneumonia	11 (22.92%)	2 (12.5%)	9 (28.13%)	0.293

### Treatment

The average time from the onset of symptoms to admission for all patients was approximately 10 days, with no difference between the two groups (*P* > 0.05) (Table 2). Antibiotic treatment was similar for all MPP patients. Before admission, most patients had been initially treated with oral cephalosporins and macrolides. After admission, they were intravenously administered with azithromycin or doxycycline (age ≥ 8 years). Methylprednisolone was administered for anti-inflammatory treatment to patients with refractory *Mycoplasma pneumoniae* pneumonia. For patients with the value of D-dimer > 5.0 mg/L, patients were subcutaneously injected with 0.01 mL/kg of low molecular weight heparin(LMWH) every twelve hours for prophylactic anticoagulation until the value of D-dimer < 5.0 mg/L. For patients with embolism, they were applied LMWH to anticoagulate for 2 weeks. Two children were used urokinase(4400u/kg/h) for thrombolysis for 3 to 5 days. Patients were treated with rivaroxaban orally until the embolism disappeared after reexamination. The detailed information of patients before and after admission is shown in Table 4.

**Table 4:** Treatment before and after admission

Case	Time from onset to admission (days)	History of treatment before admission	Diagnosed as Mycoplasma pneumoniae pneumonia before admission	Therapeutic drugs after admission
1	15	Cephalosporins and macrolide antibiotics(Oral)	Yes	Azithromycin(intravenously), LMWH(subcutaneous injection), rivaroxaban (oral)
2	6	Cephalosporins(Oral)	No	Azithromycin, methylprednisolone(intravenously) and LMWH(subcutaneous injection), rivaroxaban (oral)
3	8	Azithromycin(Oral)	No	Azithromycin, urokinase(intravenously) and LMWH(subcutaneous injection)
4	12	Azithromycin(Oral)	Yes	Azithromycin, methylprednisolone(intravenously) and LMWH(subcutaneous injection), rivaroxaban (oral)
5	18	Cephalosporins and Azithromycin(Oral)	Yes	Azithromycin(intravenously), LMWH(subcutaneous injection), rivaroxaban (oral)
6	10	Cephalosporins and Azithromycin(Oral)	No	Doxycycline, methylprednisolone(intravenously) and LMWH(subcutaneous injection), rivaroxaban (oral)
7	5	Azithromycin(Oral)	No	Azithromycin, urokinase, methylprednisolone(intravenously) and LMWH(subcutaneous injection), rivaroxaban (oral)
8	10	Cephalosporins and Azithromycin(Oral)	No	Doxycycline, methylprednisolone(intravenously) and LMWH(subcutaneous injection), rivaroxaban (oral)
9	14	Cephalosporins and Azithromycin(Oral)	Yes	Azithromycin, methylprednisolone(intravenously) and LMWH(subcutaneous injection), rivaroxaban (oral)
10	9	Cephalosporins and Azithromycin(Oral)	No	Azithromycin(intravenously), LMWH(subcutaneous injection), rivaroxaban (oral)
11	15	Cephalosporins and Azithromycin(Oral)	Yes	Azithromycin, methylprednisolone(intravenously) and LMWH(subcutaneous injection), rivaroxaban (oral)
12	6	Cephalosporins(Oral)	No	Azithromycin, methylprednisolone, mannitol(intravenously),heparin calcium(subcutaneous injection), aspirin(Oral), ganglioside, creatine phosphate sodium, rivaroxaban (oral)
13	7	Azithromycin(Oral)	No	Azithromycin(intravenously), LMWH(subcutaneous injection), rivaroxaban (oral)
14	9	Cephalosporins and Azithromycin(Oral)	No	Azithromycin(intravenously), LMWH(subcutaneous injection), rivaroxaban (oral)
15	12	Cephalosporins(Oral)	No	Doxycycline and LMWH(subcutaneous injection), rivaroxaban (oral)
16	10	Cephalosporins and Azithromycin(Oral)	Yes	Azithromycin(intravenously), LMWH(subcutaneous injection) rivaroxaban (oral)

*LMWH* Low molecular weight heparin

### Outcome

No death and recurrent thrombosis occurred in all children. Pulmonary CTA revealed that the filling defect disappeared in all children with pulmonary embolism after 1 to 3 months. Three children with cardiac thrombus disappeared after 0.5–3 months. Three children with cerebral embolism were followed up for 1 year. Limb activity of one child was better than before, however, sequelae including hand shaking and slightly poor fine movement were evident. Two children still have some symptoms, such as unclear pronunciation, unstable walking alone, reduced muscle volume, decreased muscle strength, postural tremor, shaking of holding objects, etc. MRA of the head revealed that the distal end of the branch blood vessel had not developed without a change from the previous one. The establishment of certain softening foci accompanied the cerebral infarction.

### Risk factors for embolism caused by MPP

#### The univariate analysis

The univariate analysis showed significant differences between the embolism group and non-embolism group in Tmax, CRP, D-dimer (closest to CTA/MRA), N%, pulmonary consolidation (⩾ 2/3 lobe), pleural effusion and atelectasis(*P* < 0.05).

#### ROC Curves

**Fig. 3:**
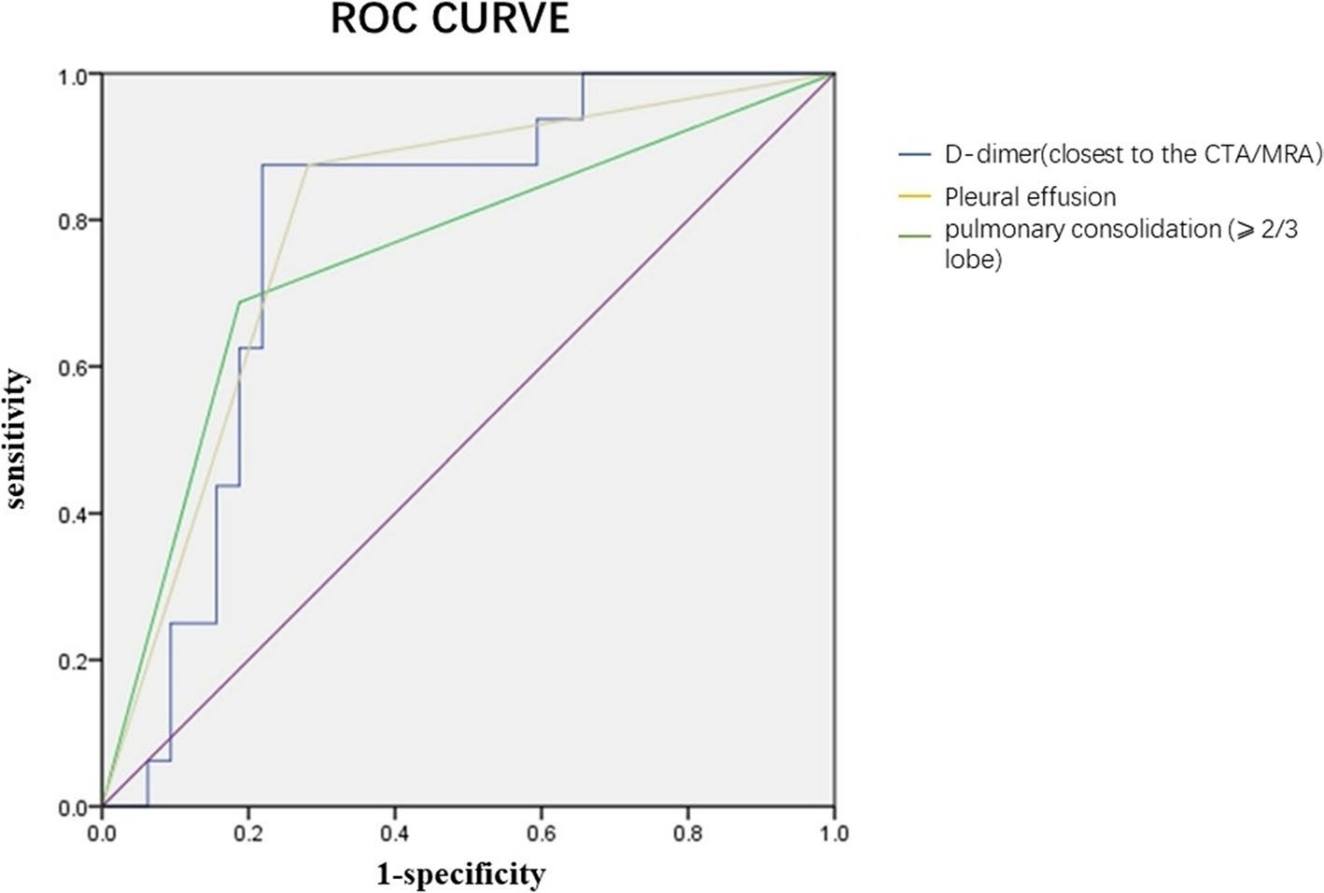
ROC curve for predictive values of the independent correlation factors of MPP with embolism in children

The ROC analysis was used to explore the predictive factors for MPP with embolism, and the critical value with maximum sensitivity and specificity was also presented in Fig. 3. ROC analysis showed that D-dimer (closest to CTA/MRA), pulmonary consolidation (⩾ 2/3 lobe) and pleural effusion were significant in diagnosing MPP with embolism. When the cut-off value for the D-dimer (closest to CTA/MRA) was set at 3.55 μg/L, the sensitivity, specificity and AUC in recognized MPP with embolism were 0.875, 0.781 and 0.781, respectively in Table 5.

**Table 5:** Predictive values of the independent correlation factors for Embolism children with MPP

Independent factors	Cutoff value	Sensitivity	Specificity	AUC	*P*-value
D-dimer(closest to the CTA/MRA)	3.55	0.875	0.781	0.781	0.002
Pulmonary consolidation (⩾ 2/3 lobe)		0.688	0.813	0.750	0.005
Pleural effusion		0.875	0.719	0.797	0.001

#### The Multivariate logistic regression analysis

Seven significant independent variables in the univariate analysis were performed for multiple logistic regression. D-dimer (closest to CTA/MRA) > 3.55 μg/L, pulmonary consolidation (⩾ 2/3 lobe) and pleural effusion played a significant role in predicting the MPP with embolism, with the odds ratio (OR) values of 1.255, 8.050, and 25.321, respectively (Table 6).

**Table 6:** Risk factors for embolism children with MPP

	B	S.E	Waldχ2	*P*	OR	95% Cl
D-dimer(closest to the CTA/MRA)	0.227	0.103	4.825	0.028	1.255	1.025 ~ 1.537
Pulmonary consolidation (⩾ 2/3 lobe)	2.086	0.914	5.202	0.023	8.050	1.341 ~ 48.327
Pleural effusion	3.232	1.135	8.107	0.004	25.321	2.738 ~ 234.205

## Discussion

Thrombosis has high morbidity and mortality, however, it is rare in children, specifically associated with infection. Notably, case reports and studies have confirmed the presence of embolism among patients with MPP [3, 6, 7]. MPP usually occurs in previous healthy school-age children. A few mild children may exhibit atypical clinical symptoms and are easily to misdiagnosed. In recent years, embolism caused by MPP has attracted more and more attention of pediatricians. In this study, we explore the clinical characteristics and risk factors of embolism in children with MPP by analyzing clinical information. Embolism induced by MPP may occur in any part of the body. Its location has previously been reported in the lungs, heart, brain, neck, abdomen and limb [3], as shown in our study(Table 1). In some patients, embolism can even develope in multiple parts. Herein, three patients (12%) had more than one distribution of embolism. One study [8] reported that the development of the cerebral infarction, abdominal organs and extremities takes 2 days to 3 weeks, 1 week to 1 month and 1–2 weeks respectively. In another study on cardiac embolism among children [9] caused by *Mycoplasma* *pneumoniae* infection, the average diagnostic time of 10 children was 13.3 days which is quite close to what we found in this research. Besides, the average diagnostic time for pulmonary embolism, cardiac embolism, cerebral embolism, splenic embolism and limb embolism were 13.4 days, 8.7 days, 15 days, 14 days and 10 days, respectively. Pulmonary embolism in adults is often characterized by chest pain, chest tightness and dyspnea, however, the symptoms of children are often atypical and easy to be ignored[10]. Sheng et al.[11] showed that all the children in MPP with embolism present fever and cough, whereas 5 (71.43%) children developed dyspnea. In our study, all children with pulmonary embolism had fever and cough. Two children (10%) presented shortness of breath and three children (30%) developed pain in various parts of the body. Only one child developed dyspnea, whereas none had hemoptysis. Children with cardiac embolism may have clinical manifestations, including chest pain, shortness of breath or complicated with pleural effusion, but without specificity [9]. In our study, children with cardiac embolism not only had shortness of breath, but also developed hypoxemia and neck pain. Children with cerebral embolism may manifest neurological symptoms, including disorder of consciousness and unilateral limb weakness [3]. In our study, limb weakness occurred in 3 children (100%) with cerebral embolism, whereas one of them had positive pathological signs and dyspnea respectively. Children with splenic embolism had left upper abdomen and periumbilical abdominal pain with vomiting (Table 1). The mechanism of embolism development in patients with MPP remains unclear. So far, relevant animal studies have not been reported. The mechanism may include the following aspects [4, 12–15]: i. MP can cause microvascular endothelial injury and release cytokines, resulting in local vasculitis and thrombotic vascular occlusion without systemic hypercoagulability. ii. MP infection promotes the body to produce autoantibodies such as anticardiolipin antibodies (ACA), β2-glycoprotein antibodies or lupus anticoagulant antibodies, and then form immune complexes, resulting in the injury of respiratory tract and other organs outside the lungs, or MP activates complement and then activates the coagulation system which may result in thrombotic vessel occlusion. Antinuclear antibody test was performed in 6 children and two children were positive (both titers were 1:100). Two children were examined for ACA and one of them had increased ACA-IgA. Protein C activity and protein S activity were performed in 6 children. Protein S activity decreased in both children, whereas protein C activity increased in one child (Table 1). After treatment, all the children returned to normal. Elsewhere, Liu et.al found that protein S activity was low in 5.1% (2/39) of patients and protein C activity was normal. ANA was positive in 51.2% (22/43) of patients [3]. In our study, half of the pulmonary thrombosis patients occurred on the same side as the pulmonary consolidation, further confirming the hypothesis that isolated arterial thrombosis may be in situ thrombosis [3]. D-dimer is a specific degradation product of cross-linked fibrin that primarily reflects blood hypercoagulability, intravascular thrombosis and secondary fibrinolysis [16]. One study has shown that D dimer can be recognized as an indicator to evaluate the severity of MPP and the incidence of extrapulmonary complications increased with the increase of D-dimer level [17]. D dimer significantly increased in the past reported cases of MPP with embolism [11, 18–20]. According to Liu et al., the average D-dimer in 10 children with cardiac embolism was 12.685 mg/L [9]. Sheng et al. found that the average D-dimer in 7 children with pulmonary embolism was 3.626 mg/L [11]. Furthermore, Liu et al.[3] found that D-dimer > 11.1 mg/L (even > 5.0 mg/L) would help in the early diagnosis of thrombosis in MPP children. However, D dimer has a high sensitivity but poor specificity, which can be increased in several diseases [4]. The results of our case–control study showed that D-dimer (closest to CTA/MRA) > 3.55 mg/L, pulmonary consolidation (⩾ 2/3 lobe) and pleural effusion were independent risk factors for embolism in children with MPP. This further confirms that the severity of MPP is the most strongly associated risk factor for MP-associated thrombosis [3]. The American Society of Hematology guideline panel developed detailed guidelines for the treatment of pediatric venous thromboembolism in 2020 [21]. The guidelines suggested ① using direct oral anticoagulants (DOACs) over vitamin K antagonists (VKAs) for patients with DVT and/or pulmonary embolism (PE), however, it may not apply to certain patients with such as renal insufficiency (creatinine clearance < 30 mL/min), moderate to severe liver disease and orantiphospholipid syndrome et.al; ② using thrombolytic therapy followed by anticoagulation over anticoagulation alone in patients with PE and hemodynamic compromise; ③ for patients with PE with echocardiography and/or biomarkers compatible with right ventricular dysfunction but without hemodynamic compromise (submassive PE), using anticoagulation alone over the routine use of thrombolysis in addition to anticoagulation (using systemic thrombolysis over catheter-directed thrombolysis); ④ using a shorter course of anticoagulation for primary treatment (3–6 months) over a longer course of anticoagulation for primary treatment (6–12 months) for primary treatment of patients with DVT and/or PE. In our hospital, all the children were applied LMWH to anticoagulate for 2 weeks. Two children were used urokinase for thrombolysis for 3 to 5 days. This study also has some limitations. Firstly, it was a retrospective study and some data were missing. Secondly, the sample size in our study is small and we need to further carry out large sample prospective studies. In addition, relevant animal studies need to be established to study the mechanism of embolism development in patients with MPP.

## Conclusions

In conclusion, children with embolism caused by MPP have higher D-dimer level and severe imaging findings. D-dimer (closest to CTA/MRA) > 3.55 mg/L, pulmonary consolidation (⩾ 2/3 lobe) and pleural effusion were independent risk factors for embolism in children with MPP. Early diagnosis and anticoagulant therapy can effectively reduce complications and mortality.

## Data Availability

The datasets generated and/or analysed during the current study are available from our manuscript.

## Abbreviations

MPP: *Mycoplasma* *pneumoniae* pneumonia
Tmax: The maximum body temperature
N%: The percentage of neutrophils
PCR: Polymerase chain reaction
VTE: Venous thrombo embolism
CVDs: Cardiovascular diseases
ACA: Anti cardiolipin antibody
ANA: Anti-nuclear antibody
BMI: Body mass index
WBC: White blood cell
N: Neutrophils
L: Lymphocyte
CRP: C-reactive protein
IL-6: Interleukin-6
FER: Ferritin
PCT: Procalcitonin
PT: Prothrombin time
APTT: Activated partial thromboplastin time
Fg: Fibrinogen
ALT: Alanine transaminase
LDH: Lactate dehydrogenase
CK: Creatine kinase
CKMB: Creatine kinase-MB
IgM: Immunoglobulin M
